# Exploring the factors that influence the decision to adopt and engage with an integrated assistive telehealth and telecare service in Cambridgeshire, UK: a nested qualitative study of patient ‘users’ and ‘non-users’

**DOI:** 10.1186/s12913-016-1379-5

**Published:** 2016-04-19

**Authors:** Erica J. Cook, Gurch Randhawa, Chloe Sharp, Nasreen Ali, Andy Guppy, Garry Barton, Andrew Bateman, Jane Crawford-White

**Affiliations:** Department of Psychology, University of Bedfordshire, Park Square, Luton, UK; Institute for Health Research, University of Bedfordshire, Putteridge Bury, Hitchin Road, Luton, UK; Norwich Medical School, Faculty of Medicine and Health Sciences, Chancellor’s Drive, University of East Anglia, Norwich, UK; Cambridgeshire Community Services NHS Trust, Saint Ives, PE27 4LG UK

## Abstract

**Background:**

There is a political drive in the UK to use assistive technologies such as telehealth and telecare as an innovative and efficient approach to healthcare delivery. However, the success of implementation of such services remains dependent on the ability to engage the wider population to adopt these services. It has been widely acknowledged that low acceptance of technology, forms a key barrier to adoption although findings been mixed. Further, it remains unclear what, if any barriers exist between patients and how these compare to those who have declined or withdrawn from using these technologies. This research aims to address this gap focusing on the UK based Cambridgeshire Community Services Assistive Telehealth and Telecare service, an integrated model of telehealth and telecare.

**Methods:**

Qualitative semi-structured interviews were conducted between 1^st^ February 2014 and 1^st^ December 2014, to explore the views and experiences of ‘users’ and ‘non-users’ using this service. ‘Users’ were defined as patients who used the service (*N* = 28) with ‘non-users’ defined as either referred patients who had declined the service before allocation (*N* = 3) or had withdrawn after using the ATT service (*N* = 9). Data were analysed using the Framework Method.

**Results:**

This study revealed that there are a range of barriers and facilitators that impact on the decision to adopt and continue to engage with this type of service. Having a positive attitude and a perceived need that could be met by the ATT equipment were influential factors in the decision to adopt and engage in using the service. Engagement of the service centred on ‘usability’, ‘usefulness of equipment’, and ‘threat to identity and independence’.

**Conclusions:**

The paper described the influential role of referrers in decision-making and the need to engage with such agencies on a strategic level. The findings also revealed that reassurance from the onset was paramount to continued engagement, particularly in older patients who appeared to have more negative feelings towards technology. In addition, there is a clear need for continued product development and innovation to not only increase usability and functionality of equipment but also to motivate other sections of the population who could benefit from such services. Uncovering these factors has important policy implications in how services can improve access and patient support through the application of assistive technology which could in turn reduce unnecessary cost and burden on overstretched health services.

## Background

The UK’s population is ageing, with nearly 11.5 million older people (aged 65+) currently living in the UK, with this figure expected to double by 2035 [1, 2]. The implications of an ageing population is marked with an increased prevalence of both chronic and long term illnesses [3]. There are over 4 million older people diagnosed as having a longstanding, limiting illness [4], which accounts for 50 % of those who are 75 years and older. At the same time the number of younger adults with a physical disability or a long term health condition is also set to rise, [5] which with the continued economic pressure on the health and social care budget [6] demonstrates a pressing need to find more innovative and cost-effective delivery models that will provide more efficient care [3, 7].

In line with the ‘Three Million Lives’ campaign there has been an increased political interest and investment in the use of assistive technologies within the UK [8, 9] through the application of both Telehealth (TH) and Telecare (TC). TH broadly refers to the use of technology to enable patients to remotely exchange clinical ‘vital signs’ information to support the management of long-term health conditions [10], for example blood pressure readings. TC is defined as personal and environmental sensors in the home to enable people to remain safe and independent in their own home for longer [9], for example alarmed medication containers. However, the success of implementing assistive technology within health and social care remains dependent on the ability to engage the general population. There is therefore a timely need to uncover the factors that facilitate or impede patient adoption and engagement [11]. In the following three sub-sections, we will explore the past literature on TC and TH adoption and the theoretical framework of this study.

### Telecare: factors influencing ‘user’ adoption and engagement

There remains limited consensus on how TC should be defined. The main issue has surrounded the use of interchangeable terminology used both nationally and internationally, with terms including; assisted-living technologies, telemedicine, telehealthcare and connected care often used synonymously [12, 13]. Further, the connotations of what TC refers to is often misplaced. For example, in the UK, TC is grounded within the roots of social care, centred on promoting safety and independence to enable patients to live in their own homes for longer [14]. However, internationally, TC is often referred to as a provision of healthcare at a distance [12, 13]. Whilst it is not in the scope of this paper to address these challenges, it is important to note that the presented research refers to two types of TC: (1) use of telecommunication where activation by the patient is needed i.e. equipment that requires users to activate the call by pulling a cord or pressing a red button and (2) passive sensors and detectors, i.e. equipment that has sensors that are triggered automatically (Table 1).

**Table 1:** Cambridgeshire Community Services (CCS) assistive telehealth and telecare service profiles

	Service profile	Description
Electronic assistive technology	Standalone	Individual pieces of electronic equipment that enhance a service user’s independence by prompting and reminding. They do not send alerts to either a carer or monitoring centre. Items include medication reminders, task prompting and orientation devices.
Telecare	Telecare standalone	Standalone telecare is similar to connected telecare. The main difference is that the sensors and detectors are NOT connected to a monitoring call centre but are programmed to link to pagers or mobile phones carried by a carer. The variety of sensors and detectors is similar to that of connected telecare and includes for example, bed and chair leaving alarms, fall detectors, epilepsy or enuresis monitors, door contact, flood, gas and smoke detectors and temperature extreme sensors. There are also proximity alarms, GPS positioning/tracking and buddy systems. The standalone telecare solutions avoid the costs associated with monitoring call centres but do require an informal or formal carer who can provide a 24-hour response to the alerts.
	Telecare connected	This equipment includes wired and wireless sensors and detectors that are programmed through a base unit telephone or call system to raise an alarm to the monitoring centre. The monitoring centre then tries to contact nominated key holders or emergency services and can provide advice and reassurance via the phone for the service user. The variety of sensors and detectors are similar to that of standalone telecare and includes for example, bed and chair leaving alarms, fall detectors, epilepsy or enuresis monitors, flood, gas and smoke detectors and temperature extremes. Activity monitoring is also possible via PIR and door monitors in the home environment or via watches or straps worn by the individual. There is normally a charge for the services of the monitoring call centre but this may be subsidised via the local authority housing services or can be subscribed to privately.
Telehealth	Telehealth connected	This involves a home telehealth monitor and peripherals for measuring vital signs that are connected via a telephone line/blue tooth and automatically transmits the data to a monitoring clinician via a secure and confidential website. The monitoring clinician reviews the trends of the readings and signs/symptoms to instigate a treatment plan to stabilise the long-term condition. The vital signs that are most frequently monitored are temperature, heart rate, blood pressure, SPO2, weight, blood glucose and the most common conditions are COPD, heart failure, hypertension and diabetes.
	Telehealth standalone	Service users take their own readings using calibrated equipment, for example, weighing scales, thermometer, blood pressure cuff or blood glucometer. The service users then manually transmit this data via e-mail, telephone or text, to the monitoring centre who record this onto a clinical system and instigates appropriate responses according to the plan made in advance. The vital signs that are most frequently monitored are temperature, heart rate, blood pressure, SPO2, weight and blood glucose and the most common conditions are COPD, heart failure, hypertension and diabetes.

TC is claimed to provide safety and high end reassurance to patients and carers [8], which in turn, supports older people and those who have a disability to live independently in their own home for longer [14]. However, evidence suggests that adoption of TC, particularly among the older population remains relatively low [15]. It is widely claimed that older people are active critics of assistive technologies, which has consequently contributed to a ‘digital divide’ in the UK ageing population [16, 17]. However, research has suggested that whilst complex assistive technologies are perceived as challenging particularly when patients have suffered cognitive decline [18], more basic technologies are deemed acceptable in older population groups [19]. Stigma of using TC equipment and its association with being ‘older’ or ‘disabled’ has been shown to negatively impact on uptake with applications viewed as a threat to patients’ identity and autonomy [20]. Thirdly, unattractive designs of TC devices and their incompatibility with ‘users’ day-to-day life are also cited as core reasons to low adoption [18].

### Telehealth: factors influencing ‘user’ adoption and engagement

The difficulty of defining TH has also been met with marked challenges. Whilst in the UK TH focuses on the delivery of remote care using equipment to transfer vital health signs, globally, TH is often used interchangeably with telemedicine [21]. This debate is made even more complex by the continual changes to improving technology and the way these devices are delivered in practice. For example, in the UK the Department of Health outline that TH readings are automatically transmitted [9], but in community settings this is often not the case. Often patients take readings at home and submit them to a service either by email, text or phone or via a mobile health application who would then enter the details into the electronic patient record. Further, the Department of Health outline that TH interventions are delivered in ‘real-time’. Whilst this is often the case in hospitals, this is less common in community based services who frequently receive patients’ vital signs readings at agreed time intervals rather than in ‘real-time’.

Despite these challenges there remains a good evidence base to suggest that TH can support patients to have a greater involvement in decisions relating to their health condition and encourage them to take increasing responsibility for their own health [9, 22], both of which are linked to increased patient satisfaction and reduced health service uptake [23]. Research has suggested that generally patients adapt well to the use of TH, with TH equipment viewed as simple and straightforward to use [24, 25], and reassurance shown to be a prominent influencer of technology adoption [25, 26]. However, the shift from traditional ‘face-to-face’ healthcare and technical frustrations shown by some patients include faulty equipment, delayed data transmission and inaccurate readings and contribute to low engagement by some sections of the population [25, 26]. Nonetheless, existing research, similar to studies based on TC adoption, have exclusively focused on current ‘users’ of TH [25, 26] rather than exploring the views of ‘non-users’. Further, there also remains a paucity of research that has identified how barriers impact not only adoption of TH and TC but also continued engagement.

### An integrated theoretical approach to adoption and engagement

The Technology Adoption Model (TAM) [27] and the Theory of Planned Behaviour (TPB) [28] have been the most extensively applied theories used to understand and predict technology adoption in healthcare [29]. Whilst most research has successfully applied these models to understand and predict physician adoption of technology [30, 31], there is less evidence of their effectiveness to explain patient adoption and engagement to both TC and TH systems [29]. The TAM, underpinned by ‘infusion system’ theory, can explain adoption of health technology through perceived ease of use and ‘technology anxiety’ i.e. the apprehension an individual may face about the use [27, 32]. The TPB has uncovered the central influence of others when choosing healthcare [32], with subjective norms and social pressure cited as prominent predictors of healthcare technology adoption [32]. Moreover, attitudes and perceived control have been identified as persuasive factors in explaining utilisation of technology-based applications [33, 34].

The Unified Theory of Acceptance and Use of Technology (UTAUT) [35] presents a revised model, integrating both the TPB and TAM to understand and predict end user acceptance of technology [36, 37]. The UTAUT suggests adoption is impacted by four factors: (1) performance expectancy i.e. technology will enhance quality of life performance; (2) effort expectancy i.e. ease of use; (3) social influence i.e. views of important others towards the use of the new technology and finally, (4) facilitating conditions, which refers to an individual’s belief that the organisational and technical infrastructure exists.

Current theoretical understanding of TH and TC is still in its infancy. Many studies have focused on adoption of different types of technology ranging from mobile phones applications to telemedicine systems. This is complicated by the differing TH and TC devices operated globally, which are delivered in very different healthcare settings. There is also limited understanding as to how other influential factors impact on adoption of TH and TC. For example, concepts borrowed from the Health Belief Model (HBM) [38] such as knowledge, perceived benefits and barriers, and individual perceptions i.e. susceptibility and severity of symptoms have been shown to directly impact on decision making for heath care service uptake [39, 40] and more recently applied to explain TH technology adoption [41]. Outside of this, there is limited theoretical understanding of what factors impact on continued engagement once patients adopt these services.

### Rationale and aims

There is an increased political interest and investment in the use of assistive technologies within the UK [8, 9] through the application of Telehealth (TH) and Telecare (TC). Therefore, there is a clear need to understand how patients adopt and engage in TH and TC applications to ensure that services are equitable and accessible to all [11]. Research has uncovered that adoption of TH and TC has centred on acceptance of need, perceived benefits of technology [18, 42] alongside ‘ease of use’ [20] although findings have not been consistent. Moreover, it is unclear if barriers and facilitators to adopt and engage in assistive technology differ between ‘users’ and ‘non users’ i.e. those who have declined or withdrawn from service. In light of this, the present study explored the underlying factors that impact on patients’ decisions to initially adopt and continually engage in TH and TC applications within the context of the Cambridgeshire Community Service (CCS) NHS Trust Assistive Telehealth and Telecare (ATT) service, a service model of TH and TC to support people with health and social care needs through an integrated care service [43].

## Method

### Setting

The research took place in CCS ATT service based in Cambridgeshire, UK. The ATT service provides support to the population in Cambridgeshire who have a wide range of conditions in particular, dementia, memory impairment and head injuries; long-term health conditions (e.g. asthma, diabetes, coronary heart disease and Chronic Obstructive Pulmonary Disease COPD) and progressive conditions (e.g. Parkinson’s, Multiple Sclerosis). This service also supports both adults and children with a range of diagnosis including; epilepsy, acquired brain injury, learning disabilities, alongside frail older people and those receiving end of life care. The largest referrals relate to falls risk, personal safety and medicines management.

Cambridgeshire has an estimated population of 635,100 [44]. Over 16 % of the population are aged 65 or over, and 4800 residents were aged 90 or over in 2011, representing a 41 % increase since 2001 [45]. Currently, ATT service supports around 2500 people across Cambridgeshire with an expenditure on equipment and staffing for TH and TC services at £431 K in 2011–12. Recent ATT referral figures have demonstrated a 500 % increase over the past 10 years. For example, referrals made in 2003–04. 2006–7 and 2011–12 and 2014/15 referrals increased from 210, 398, 1154 to 1601 respectively. Referrals in 2014/15 to TH and TC services demonstrated that whilst adoption is distributed across a broad range of service user groups, older age groups (70 years+) remain the largest user category accounting for 75 % of all referrals.

The ATT service is an integrated health and social care service and completes patient assessments and issues equipment on behalf of both statutory bodies. The ATT service works within the context of wider integration of health and social care services with locality based teams of social workers, district nurses, care coordinators, community therapists and multi-skilled assistants focusing on the needs of adults and older people. This integrated model of service delivery is able to support patients with multiple needs across health and social care by allowing patients to receive equipment from more than one service profile. Moreover, the ATT service can be more adaptable and responsive to any changes of a patient’s needs where the service provision can be upgraded or downgraded as needed. In line with the NHS Act 1977 [46] and the NHS and Community Care act 1990 [47] all NHS and community equipment or any minor adaptations that cost £1000 or less cannot be charged for either by local authorities or the NHS [48].

The ATT service provides a range of electronic technological devices to support the population in Cambridgeshire and their carers to address challenges to everyday living and enhance patient independence. Similar to the Whole System Demonstrator (WSD) [49], the ATT service operates four basic service profiles including ‘telecare connected’, ‘telecare standalone’, ‘telehealth connected’ and ‘telehealth standalone’. However, the ATT service provides a fifth additional ‘standalone’ profile. The ‘standalone’ profile is the application of simpler, cheaper standalone device/s that are not linked to another device/monitor and are solely operated by either the individual or carer; examples would include simplistic medication reminders and key finders.

The ATT service does not operate to a condition specific protocol i.e. ‘if you have this condition you need this equipment’. Instead, assessments are based on individual needs including functional and cognitive ability to operate equipment and the social circumstance of availability of a carer to help. Some equipment can also be standalone as well as connected, for example automatic medication reminders can be standalone or can be connected to a lifeline device, a 24-h personal alarm. Assistive devices provided by ATT service are not intended as a sole solution but a tool to supplement and support other services provided by professionals, family and carers.

### Participants and methods

In-depth, one-to-one, semi-structured interviews were conducted from 1^st^ February 2014 until 1^st^ December 2014 (outside of the service evaluation period) to explore views and experiences with both ‘users’ and ‘non-users’. Participants were recruited through the ATT service, who were referred to use one or more of five service profiles (1) ‘telecare connected’, (2) ‘telecare standalone’, (3) ‘telehealth connected’, (4) ‘telehealth standalone’ and finally (5) ‘standalone’ (see Table 1).

#### ‘Users’ (patients)

‘Users’ for the purpose of this paper refers to either current or existing patients who had used the ATT service during the evaluation period, which was from the time period 1^st^ August 2013 and January 31^st^ 2014. Participants were purposefully selected for sample variation across five service profiles (telehealth standalone, telehealth connected, telecare standalone, telecare connected and standalone only) and demography. Participant details of ATT patients who were included in the study (*N* = 28) are presented in Table 2. Ages ranged from 35–92 (*M* = 67.0; SD = 14.5) with the majority of ‘users’ telecare and standalone (*N* = 23) and the remaining ‘users’ of telehealth connected (*N* = 2) and telehealth standalone (*N* = 3) service profiles, which demonstrated a similar distribution to the ATT service utilisation.

**Table 2:** Participant details of ‘users’ patients who adopted and engaged in the service

Participant	Service profile	Gender	Age	Medical condition	Equipment
Beatrice	Telecare- connected	Female	62	Epilepsy	Pendant and Pager, wrist worn fall detector
John	Standalone	Male	69	Parkinson’s disease	Pivotell medication reminder with dispenser, large dossett box
Roger	Standalone	Male	75	Parkinson’s disease	Wrist worn medication reminder
Thomas	Telecare- standalone	Male	70	Parkinson’s disease	Dossett Box, Wrist worn medication reminder, Pendant and Pager
Alice	Standalone	Female	76	Risk of falls	Pivotell medication reminder and dispenser
Penny	Telehealth- standalone	Female	74	Chronic obstructive pulmonary disease	Telehealth: Temperature and pulse (standalone)
Tim	Telehealth- standalone	Male	67	Chronic obstructive pulmonary disease	Telehealth- Temperature and pulse (standalone)
Henry	Telecare- standalone	Male	90	Stroke	Mobile Phone-Tracker
Marie	Standalone	Female	59	Physical disability	Pill reminder
Louise	Telecare- standalone	Female	35	Epilepsy, myalgic encephalomyelitis	Pendant and Pager
Andrew	Telehealth- standalone	Male	66	Chronic obstructive pulmonary disease	Telehealth- temp and pulse (standalone)
Loretta	Telecare- standalone	Female	66	Multiple sclerosis	Pendant and Pager
Kelly	Telecare- connected	Female	39	Epilepsy	Pendant and Pager, Smoke alarm (with lifeline)
Cathy	Telecare- connected	Female	53	Epilepsy	Wrist worn epilepsy sensor
Sheila	Standalone	Female	47	Heart attack	Pivotell medication reminder and dispenser
Tracey	Standalone	Male	46	Irritable bowel syndrome/depression	Pivotell medication reminder and dispenser
Mary	Telecare- connected	Female	85	Epilepsy	Fall detector, bed sensors
Philip	Standalone	Male	92	Risk of falls, cognitive impairment	Pivotell medication reminder and dispenser
Carole	Telecare- standalone	Female	49	Physical disability	E-Pill reminder
Helen	Telecare- connected	Female	79	Cerebral palsy	Wrist worn fall detector
Grace	Telecare- standalone	Female	78	Multiple sclerosis	Pendant and Pager
Norma	Telecare- connected	Female	85	Risk of falls	Bed and chair leaving alarm, wrist worn fall detector
Gloria	Telehealth- connected	Female	72	Chronic obstructive pulmonary disease	Connected telehealth - temperature, pulse, SPO2
Susan	Telecare- connected	Female	70	Parkinson’s disease, risk of falls	Bed leaving alarm, pendant and pager
Clive	Telecare- standalone	Male	73	Head injury	Memominder
Steve	Telehealth- connected	Male	66	Chronic obstructive pulmonary disease	Weight, blood pressure and SPO2 and questionnaire
Irma	Telecare- connected	Female	71	Risk of falls	Fall detector
Howard	Standalone	Male	62	Parkinson’s disease	Pendant and Pager, wrist worn fall detector

#### ‘Non-users’ (withdrawn/declined)

‘Non-users’ is an umbrella term to describe two sets of patients. First, patients who declined to use the ATT service after they were referred, and second, patients that had received ATT equipment but subsequently withdrew. Reasons for withdrawal and declination were not captured. All ‘non-users’ were referred to the ATT service during the service evaluation period, during 1^st^ August 2013 to 31^st^ Jan 2014. The ATT team sent participant information letters to all participants who met the inclusion criteria (*N* = 113) to invite them to take part in this study. A total of 39 % (*N* = 44) replied, with 12 agreeing to take part in the study.

Patients who declined the service after referral alongside those who agreed to be referred then withdrew from the service were both included in the study. Those who declined the ATT service before being allocated to a service profile provided a rich deeper understanding of the perceptual aspects which impacted on their decision to not use the service. From the total sample, 3 of the 12 participants declined at referral stage and were not allocated to a profile. The remaining ‘non-users’ (*N* = 9) were referred into the ATT service but then withdrew after equipment installation. Whilst both groups of ‘non-users’ were combined to achieve saturation, their distinction is made clear in the findings. Ages of participants ranged from 24–92 (*M* = 63.3; SD = 21.47). Across the service profiles, ‘non-users’ were predominantly telecare and standalone service patients (58.3 %), with the remaining ‘non-users’ allocated to the telehealth standalone service profile (Table 3).

**Table 3:** Participant details of ‘non-users’ who have withdrawn/declined

Participant	Service profile	Gender	Age	Medical condition	Equipment
Barry	Telecare standalone	Male	59	Stroke	Pendant and pager
Arthur	Telehealth standalone	Male	92	Chronic Obstructive Pulmonary Disease	Telehealth: Pulse & temp
Casey	None^a^	Female	32	Myalgic Encephalomyelitis	None^a^
Joan	Telecare standalone	Female	49	Multiple Sclerosis	Pendant and pager
Jean	Telecare standalone	Female	82	Falls	Pendant and pager
Margaret	Telehealth standalone	Female	75	Chronic Obstructive Pulmonary Disease	Pulse Oximeter
Ken	Standalone	Male	66	Parkinson’s	Wrist worn medication reminder
Jim	None^a^	Male	52	Pancreatitis	None^a^
Ian	Telehealth- standalone	Male	69	Chronic Obstructive Pulmonary Disease	Oxygen and temp equipment
Edith	Standalone	Female	92	Congenital palsy osteoporosis	Dossett Box^a^
Della	Telecare- standalone	Female	68	Parkinson’s Disease	Medical arm
James	Telecare-standalone	Male	24	Brain injury	Bed leaving alarm kit

^a^ Patient decined service before allocation to service profile/equipment

### Interview process

#### Recruitment and sampling

**Fig. 1:**
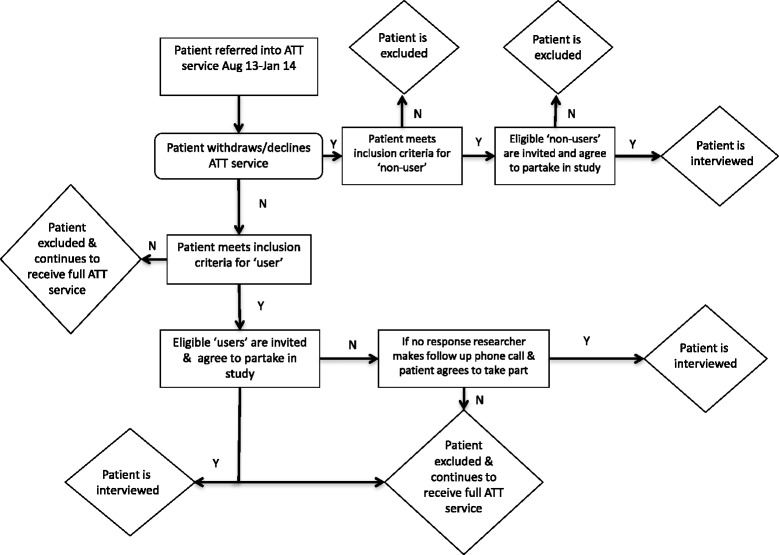
Recruitment pathway for ‘users’ and ‘non-users’

The ATT team posted participant information letters to all participants who met the inclusion criteria i.e. referred within the service evaluation period of 1^st^Aug 2013 and 31^st^ Jan 2014. All children were excluded (aged <18) alongside anyone who lacked mental capacity to consent. The invitation letter asked potential participants to state if they were either a) interested in taking part in the study and being interviewed, b) if they did not want to take part and c) wanted more information. If they replied, asking for more information a more detailed information sheet was posted out to them, with a follow up phone call. All participants who opted not to take part in the study were not contacted again and were immediately excluded. In situations where no response was received the interviewer phoned all potential participants to ask if they would be interested in taking part (Fig. 1). The interviewer (CH) then contacted all participants who agreed to be interviewed where an interview was arranged on a day/time which suited the patient. All interviews with patients were conducted in the environment of their choice, which was commonly within the patient’s or the carer’s home.

#### The interview guide

The interview guide was developed collaboratively as part of the multi-disciplinary research team and validated with members of the Trusts Patients Forum (TPF) and a patient experience group who included 8 non-expert public members set up in the initial stages of the project who had knowledge of TC and TH, and/or had experience of informal care. Interview questions on the decision-making process for engaging and not engaging with the ATT service were theoretically driven based on the integrated (UTAUT) [35] and the HBM [38] alongside current literature on understanding adoption and engagement behaviour.

The interview guide used open-ended questions to explore patient experiences of the ATT service, more specifically the referral process, needs assessment, installation of equipment and after care. Questions examined patient’s knowledge and awareness of the ATT service and the equipment available, attitudes towards the equipment/service as well as perceived need and confidence to use the equipment. Perceived benefits and barriers of using the equipment/service were also explored.

### Framework method

Framework Method [50] was used to analyse the data, a method that has been extensively applied to multidisciplinary health research [51–53]. This method is a grounded and generative analytical procedure which uses distinct and interconnected stages which allow the researchers to move back and forth within the data until a consistent account emerges [50, 54, 55]. The defining aspect of Framework Method is a matrix output of summarised reduced data, which allows comparisons of data to be made both across cases, in this context ‘users and ‘non-users’ as well as within individual cases [53].

The transcription of all interviews was outsourced to an independent professional transcriber. EC reviewed the first five transcripts to ensure that they were correctly formatted and that they were all consistent. Content was more important as opposed to the structure of participants’ responses for analysis, as such only long pauses, interruptions and nonverbal communication were noted within the transcript. The interviewer (CH) checked all transcripts for errors by listening back to the audio recording and reading the transcripts simultaneously.

As EC and CS had not conducted the interviews it was essential that they became immersed in the data. This familarisation process involved listening to audio recordings of the interviews alongside reading the accompanying transcripts across a range of interviews. Both researchers made analytical notes throughout, focusing on their thoughts and impressions of the narrative.

EC and CS independently coded 6 transcripts (‘users’, *N* = 3; ‘non-users’ *N* = 3). This process involved paraphrasing what was interpreted in the passage as important. Open coding was completed which involved coding anything that could been seen as important. Coding varied from small sections of data (parts of sentences) to whole paragraphs and was inductive. Written notes and ideas were also assigned to the coding label applied.

The analytic framework was developed through discussion of the codes which were assigned to each passage. The codes of data were then grouped together into categories to apply to the rest of the transcripts. EC and CS independently applied the analytic framework to two transcripts to ensure the initial framework captured the core and sub-core themes and to identify if any new open codes had emerged. The analytic framework was then applied by indexing the subsequent transcripts by EC using NVIVO v10 for Windows. This version software was used as it had the capability to generate framework matrices through the development of a matrix by participant characteristics and core and sub-core themes [56]. The matrix allowed for effective data retrieval and a high level of familiarity with the data [53]. A spreadsheet was then used to generate the matrix from the analytic framework that had been already coded, participants were put into rows and codes were shown as columns.

The data were charted into the matrix, which involved summarising, by category for each transcript. EC charted all transcripts with a sub section moderated by CS to ensure that the summary still stayed close to the words of the participant. The final stage, interpretation, involved exploring the range and diversity of coded data by refining initial themes and categories. Abstract concepts were developed collaboratively through the identification of key dimensions of the synthesised data and making associations between themes and concepts.

There were two core themes found; (1) ‘Decision to use (to not use) the ATT service and

(2) ‘Engagement and use of the ATT service’. The first theme was defined as the process that the patient goes through that in turn impacts the decision for the patient to use the ATT service. Decision making in this context was viewed as a reasoning process, which can be rational or non rational, and can be based on explicit or tacit assumptions. The second theme was defined as the benefits and barriers that impact on the decision to continue to use the equipment supplied by the ATT service.

The core themes and sub-themes were discussed with the interviewer to determine if CH considered these to be an accurate reflection of the interviews. No discrepancies were found.

### Ethics, consent and permissions

NHS ethical approval was obtained by the NRES Committee East of England (REF: 13/EE/0362) in January 2014. Each participant was provided a participant information sheet, which clearly explained the nature and purpose of the research. Informed consent was obtained through completion of a consent form, which gave permission for anonymised narratives and non-identifiable clinical information to be published. All qualitative data (including audio recordings) obtained from the interviews was stored anonymously and were destroyed after they were collaboratively confirmed as being reflective of the interview. Only EC and CS accessed anonymised transcripts where no other persons had access to the data. In the reporting and publication of the findings, where comments and quotes were used, pseudonyms were reported to maintain anonymity. The names used were purposefully chosen to reflect the ethnicity, age and gender of the participant interviewed. All participants were given a £20 high street voucher as a goodwill gesture. No transcripts were withdrawn by the participant.

## Results

The present study was interested in exploring the factors that affected patient decisions at the point of referral for both TC and TH, which ATT provides. These will be presented by core theme; Theme 1: Decision to use (or to not use) the ATT service and Theme 2: Engagement and use of the ATT service. Similarities and differences between ‘users’ and ‘non-users’ patient groups are discussed where relevant (Table 4), with example cases to illustrate sub-core themes.

**Table 4:** Overview of similarities and differences of barriers/facilitators across the participants for uptake and engagement

Theme	Sub-theme	ATT service factors ‘Users’	ATT service factors ‘non-users’
Decision to use the ATT service at point of referral	Acceptance of old age/health condition	• Most ‘users’ accepted they had a need for equipment • Perception that equipment symbolised a transition to ‘getting old’	• Most ‘non-users’ did not accept they had a need for equipment • Many non-users stated they went along with the referral to please others
	Previous knowledge & awareness of service & equipment	• Only two users had heard of ATT service before referral • Previous knowledge related to knowing of others who had used the service • Main source of information was ‘referrer’ who was most commonly a community health care professional supporting them with specialist care • Users’ often discussed the decision to use the service with the referrer, often a healthcare professional before making the decision • Had a preference for receiving information about service face-to face • ‘Users’ generally felt in charge of the decision to use the service	• None of the non-users had heard about ATT service before referral • Main source of information was ‘referrer’ who was most commonly a community health care professional supporting them with specialist care • Had a preference for receiving information about service face-to face • Some ‘non-users’ felt that they did not have enough information to make an informed decision and some feeling of being ‘pressurised’ • Many ‘non-users’ went along with the referral to keep the referrer happy, particularly their healthcare specialist
	Perceived usefulness of equipment	• “Users’ perceived that the ATT equipment would be useful • TH equipment was viewed as useful to monitor health • TC equipment perceived as useful to communicate with carer, remind them to take medication, to get help in an emergency	• Non-users did perceive the equipment as useful or feel that it would add any value
	Attitudes and perceptions towards ATT equipment	• Many ‘users’ felt that they would find the equipment easy to use and felt that they had the confidence to use it. • Older patients demonstrated more apprehension than younger ‘users’	• Many older patients lacked confidence and experience to use technology • Viewed equipment/technology as time consuming • Equipment was viewed as complex and difficult to use • Concerns around functionality and/or support with functionality from service
Engagement and use of ATT service	Usability	• Users overall found equipment relatively easy to use and set up • Pendant and pager, fall detectors and TH equipment viewed as easy to use • There were some difficulties for older patients who were using medication reminders and changing batteries • Some ‘users’ fund instructions difficult to follow	• Found equipment difficult to use and/or difficult to set up • TH users found equipment inconvenient e.g. readings at a set time • Some TC users found equipment time consuming and inconvenient e.g. stocking up medication reminders • Instructions difficult to follow
	Actual usefulness of equipment	• Most participants felt that the equipment was suitable to meet an unmet need • TC users found equipment useful and met an unfulfilled purpose • TH users felt equipment was useful as it enabled them to monitor their health and check readings were within the set parameters	• Majority of non-users stated that they did not find the equipment useful and was cited as a core reason for non-engagement • Reasons related to no perceived need or a change of need • Some felt equipment did not meet specific requirements
	Functionality of equipment	• TH users felt equipment was reliable and were confident it would work as intended/readings were accurate • TC users felt confident equipment would work in an emergency • Some issues surrounded equipment functionality but valued instant support	• Concerns relating to functional equipment • Felt unsupported to deal with technical issues • Unsure what to do when equipment was not functioning properly
	Threat to identity and independence	• Perceived stigma to using equipment in public • Acceptance of getting older or that life cannot continue the same	• Concern about loosing independence and being dependent on others

### Theme 1: decision to use (or not use) the ATT service at point of referral

This core theme captured the barriers and facilitators to using the ATT service at the point of referral. Within this core theme, there were four sub-themes that emerged: ‘acceptance of old age/health condition’; ‘previous knowledge and awareness of the service and equipment available’; ‘perceived usefulness of equipment’ and ‘attitudes and perceptions towards ATT technology’.

#### Acceptance of old age/health condition

The ‘acceptance of old age/health condition’ between TC and TH differed. For TC, the decision to use the ATT service was found to be commonly affected by the acceptance of getting older. For TH, acceptance of declining health or newly diagnosed condition impacted decision making. Hence, for patients to use either TC or TH technologies there needed to be a perceived need based on the individual’s acceptance of their health condition or older age.

It was commonplace across TC and TH ‘users’ that there was a need to come to terms with ‘getting old’ before there was an acceptance for help:

*‘It is actually coming to terms with getting old I think, before you can ask for help you have got to accept that you need it.’ (Sheila, 53, Standalone ‘user’).*

The ATT service was perceived to be a service for older people that often had negative connotations i.e. losing independence by not being able to do things they once had done and being more dependent on others:

*‘I thought that it would make out that I am old. You see up here (points to head) I am still 17 but the body, well the body is giving way.’*

*(Susan, 70, Telecare connected, ‘user’)*

*“It makes you feel old. It challenges your independence and having to rely on people all the time because stupid little things that you could do before…I was going I don’t need anything.’*

*(Mary, 85, Telecare connected, ‘user’)*

Discussions also centred on coming to terms with a health condition. Kelly was recently diagnosed with Epilepsy and she disclosed the challenges she had in accepting that she had Epilepsy. Kelly, refers to her condition, as a ‘disability’, which she had to come to terms with, and perceived the equipment to be a ‘constant reminder’: *‘A big thing for me was getting my head around the fact that I have a disability I don’t want, because you know it’s a constant reminder even when you’re well, you’re still constantly reminded that actually you’re not always well’* (Kelly, 66, Telecare connected, ‘user’).

#### Previous knowledge and awareness of service and equipment

There was a lack of awareness of the ATT service across ‘non-users’ and ‘users’ prior to referral. There had been no ‘non-users’ who had previously known about the ATT service before they were referred. Moreover, only two ‘users’ stated that they had heard about the service before they were referred. In these two instances, the patients had either used the ATT service previously or had known of others who had used the service.

Many of the participants appeared to be more aware of the types of equipment available. In particular, many ‘users’ discussed being aware of the lifeline products, although were not sure in most instances if they were part of the ATT service or who they were provided by. Further, across all participants there was a clear lack of knowledge of the service profiles and the range of equipment available before the referral was placed.

Most ‘users’ recalled receiving an ATT leaflet, to learn more about the service prior to referral. The leaflets had been obtained from the ATT service directly or through a referring agency such as a GP. These leaflets were viewed as easy to read, useful and informative:

*‘They did send me some leaflets to describe the assistive technology stuff…and they had clearly written them themselves and put in some clip art to help you through which was nice. Errm, and I felt that they were plain English type language, so you know not perhaps working to the maximum of my intellect, but something that everyone would understand you know pretty straight forward and user friendly, it was good to have something with a telephone number’*

*(Louise, 35, Telecare standalone, user)*

There was a feeling of being bombarded with leaflets and written information. On some occasions, ‘users’ and ‘non-users’ would receive a large amount of information following hospital discharge and so they found it difficult to read all of the literature:

*‘Maybe I got a leaflet, you see when you get your hospital letter there are loads of leaflets saying about this thing and that thing I would much rather talk to someone I haven’t got the patience to go through all of that’*

*(Jim, 52, declined ‘non-user’)*

In these instances, both ‘users’ and ‘non-users’ showed a higher preference for a face-to-face discussion about the service with their health care professional to learn about what the service is and how they could potentially help them.

The source of information predominantly came from the referrer, a professional who worked within housing, social and healthcare. The most common source of information included community healthcare specialists e.g. physiotherapist, Parkinson’s clinical nurse, consultant, Multiple Sclerosis clinical nurse, rehabilitation clinical nurse and Occupational Therapist (OT). Other sources of information about the ATT service came from social services, social workers, Learning Disability Partnership Team (LDPT), community matron, and charities such as Age UK.

Patients are able to also self-refer, although none of the interviewed sample did this. Instead, ‘users’ made their decision following discussions with the referrer who was either the healthcare professional or their family. For TC applications, some patients had to get confirmation from others that they could be a contact in an emergency and would discuss the referral with them also. Nonetheless, there was a general feeling across ‘users’ that they were ultimately in charge of the decision to use the service:

*‘It was me, it was solely me because I thought, I thought about everything and if there was something wrong that I couldn’t get to the bottom of then I thought it would help’*

*(Gloria, 72, Telehealth connected, ‘user’)*

Four ‘non-users’ stated that they were not aware of who referred them, and the first time they had heard about the ATT service was when they received a phone call from the ATT service to discuss a home visit. The other remaining ‘non-users’ disclosed that they went along with the referral to please the health care professional who referred them with many ‘non-users’ feeling like they had been pushed into the decision.

*‘I had a nurse come and see me about every three months, she has done for many years now and then she suggested that it might be helpful because I said I wasn’t taking my pills rigidly. She said we can get over that by giving you a beeper so it was just a case of doing what she said really’*

*(Ian, 69, Telehealth standalone, withdrawn ‘non-user)*

*‘If someone is enthusiastic about it I have to say yes because I am not one of those, I just accepted it is a good idea at the time to please the person who suggested it’*

*(Della, 68, Telecare standalone, withdrawn ‘non-user’)*

#### Perceived usefulness of equipment

This sub-theme is defined as the level of usefulness of the equipment to meet the patient’s need at the point of referral prior to using the equipment. Within this sub-theme, there were a number of factors that were discussed about usefulness including: safety of the patient; improving daily life of patient/carers/other; improve health and well being of patient/carers/others; providing reassurance to patient/carers/others and/or increasing independence for the patient.

‘Users’ generally agreed that the ATT equipment would be useful. For TH equipment, many saw the benefit of being able to monitor their own health so they would know, for example, if their oxygen levels were within the parameters. Other reasons that ‘users’ perceived the service as useful included: a reminder to take medication and to turn off electrical devices to improve household safety; communication with to carer when they required assistance and, immediate access to help in an emergency. However, the perception of the usefulness of equipment was not supported across ‘non-users’. For example, Ken withdrew from the service after being referred because he did not see the benefit of the equipment (a pendant and pager) when he already had a telephone, which in his opinion was better because you at least he could speak to his carer on the telephone*: ‘I just assumed that I wouldn't need it, I’ve got a phone if I need to ring someone I just telephone’* (Ken, 66, Standalone, withdrawn ‘non-user’).

Another patient, Casey, was referred to the TH service by her GP to have her blood pressure monitored daily. Although Casey was referred, she could not see the value this would bring. In her mind, she already had her blood pressure taken when she attended routine checks and this was enough. Therefore, she was not sure what extra information doing her blood pressure daily would provide: *‘I go and have my blood pressure taken once a year at the surgery and you know I’ve been satisfied with that, I couldn’t see the point, as far as I was concerned I couldn’t see any personal benefit…. it wasn’t going to make any difference to my life’* (Casey, 32, declined ‘non-user’).

#### Attitudes and perceptions toward ATT equipment

This sub-theme refers to the attitudes and perceptions patients had towards the equipment prior to referral.

Many ‘users’ and ‘non-users’ were unaware about the service prior to referral; therefore, perceptions towards the equipment were formed either from discussions with the referrer, written material provided to them and discussions with others (healthcare professionals/family/friends) who may have used similar devices in the past. There were very few ‘user/non-user’ patients who had seen the equipment demonstrated and therefore many did not know what the equipment was going to be like.

ATT uses a wide range of equipment, which is often described as ‘assistive technology’ or TC and TH by healthcare professionals and ATT service professionals. However, some ‘user/non-user’ patients felt that the name initially was off putting, and in hindsight was not representative of the service itself. For example, one patient discussed that the name TH and TC was not a good description as this suggested that it has connotations with telephones and health although this was not the case as they used a digital timed medication reminder. Many other participants suggested that the name assistive technology also did not effectively define or describe the service. The word ‘technology’ suggested to the participants that the technology was computer based and argued that the equipment is not what could be widely perceived as ‘technology’.

Positive attitudes centred on the perception of the ease of assistive technology use and the perceived confidence the patient had to use the equipment. There were discussions of apprehension and dislike towards technology across the ‘non-users’ who felt they lacked the confidence and experience to use technology. Many ‘non-users’ expressed a clear dislike and an overall negative attitude towards technology.

Edith for example, was referred to the ATT service following a referral from her Parkinson’s nurse to be issued an automatic medication reminder to prompt her to take her medication. After the referral and discussions with the ATT service, Edith discusses how she declined to use the equipment. She believed the equipment would be difficult to use, inconvenient and would not work or would not be supported if it did not work. This negative attitude towards technology was representative of many of the older ‘non-users’: “*Cause I am old and awkward and I thought I couldn’t be bothered, just couldn’t be bothered to be honest. I don’t want to learn how to work it they (ATT service) told me I have to put tablets in who is going to be bothered to muck around doing that every five minutes, and what about when it doesn’t work, no thanks, I don’t want to know’* (Edith, 85, Standalone, ‘declined non-user’).

### Theme 2: engagement and use of the ATT service

The reason for engagement of the ATT service focused on four sub-themes: ‘usability’, ‘usefulness of equipment’, ‘functionality of equipment’ and ‘threat to identity and independence’.

#### Usability

Usability refers to ‘ease of use’ and ‘convenience’. The majority of ‘non-users’ viewed the equipment difficult to use and/or set up, and felt increasingly frustrated trying to get the equipment to work. Whilst initially, most ‘non-users’ perceived the equipment would be relatively easy to use, this was not reflected in their experience. One example is James, who disclosed his increasing frustration when his bed sensor malfunctioned in the middle of the night, which meant that there was a loud buzzing sound, which could not be turned off despite many attempts from both the patient and their carer. This ultimately led to the decision to give back the equipment and subsequently withdraw from the service: *‘In the middle of the night I couldn’t turn it off, i’d taken the batteries out and it was still going. I must have turned it off but I don’t know, all I know was I was in bed and I was trying to get this thing with this American accent to turn off. “Oh” I just thought “I can’t do this”, it was hard to turn off…I remember just saying “it is too difficult to use and gave it back”’* (James, 24, Telecare standalone, withdrawn ‘non-user’).

However, this finding was not unique to ‘non-users’ as many ‘users’ who used the TC strand of the service discussed an element of frustration setting up the equipment;

*‘It was a pig, it really was hard work, and I mean you have got to have a science degree to work it’*

*(Thomas, 70, Telecare standalone, ‘user’)*

*‘I couldn’t open the damm thing. I still can’t but I can do the rest now, but this is really confusing, err button one and then when you carry on it doesn’t make it clear that you always use button one first and then onto the others, I think that’s how you do it but I’ll know when I open it and do it. But it’s not really clear that that’s what you do’.*

*(Tracey, 46, (Telehealth/Telecare) Standalone, ‘User’)*

A common difficulty across ‘users’ and ‘non-users’ surrounded changing batteries. Some patients were provided equipment with used batteries, and others spoke of difficulties in not knowing they had to change batteries i.e. what the warning sign was and what size battery they had to replace it with. It was evident that there was limited information provided about the upkeep of the equipment:

*‘Well you know I did find that I was coping with it until the batteries went and then it’s just been frustration ever since’*

*(Marie, 59, Standalone, ‘user’)*

The findings suggested that there were some types of equipment, which were found to be easier to use across the sample. For example, many of the ‘users’ found the pendant and pager, TH equipment and fall detectors relatively easy to use:

*‘It is easy yep, it is easy, just having to ring up, either somebody takes your readings when you ring up you get the telephone message after the tone, you leave your readings and they take it from there so it’s very straight forward. I mean if you can phone you can use the service can’t you it’s not rocket science’*

*(Andrew, 66, Telehealth standalone, ‘user’)*

*‘To be honest it’s a bit obvious it just rings through to the lifeline and then with the fall thing you know it’s obvious it will detect if you fall, that’s why it’s called a fall monitor, so you know it’s not rocket science’*

*(Kelly, 66, Telecare connected, ‘user’)*

*‘It’s not complicated, it literally tells you what to do. It tells you to stand on the scales, press this, answer your questions, you press yes or no, you know it’s a no brainer, any sort of 6 or 7 year old could do it you know’*

*(Steve, 66, Telehealth connected, ‘user’)*

However, difficulties were found across the sample using medication reminders. Many participants had to depend on the instructions, which were not clear. The medical reminders had a variety of functions and buttons to press to set the times to make sure that it corresponded to the correct medication at the correct time.

Roger, aged 75, discusses his difficulties setting up a watch pill timer, which was provided to him to help remind him to take his medication. Roger was provided support with set up although this was only *‘half done’* and despite numerous attempts to finish off the set up he eventually had to ask his son to set it up for him: *‘To set it you have to be pretty determined to sort it out, that’s the only barrier. It would appear to be quite difficult to set up. I mean when the staff brought it round she sat there for half an hour fiddling with it and she said, “Well I’ve done half of it, so I said leave with me and I’ll take it”, not knowing how difficult it was…Its resetting how it works, to go through getting it wrong and getting it wrong and keep restarting I don’t know how many people would bother’* (Roger, 75, Standalone, ‘user’).

Some ‘user/non-user’ participants raised issues about the design of the equipment. For example, Pamela, an informal carer set up the memominder for her mother but found it difficult to turn the item on without resetting it. There were other respondents who discussed the physical limitations of their condition on using some of the equipment, reducing its ease of use:

*‘It is difficult to use for people like me with Arthritis I mean, it’s [the button] quite small and on occasions I have had to press it three times. I had to get my nail inside it you know to actually push it so it is difficult’*

*(Mary, 85, Telecare connected, ‘user’)*

*‘It would be better to have a button than a thing to push, you have a lot of pressure going on your thumb and fingers, see I can’t bend my fingers…I could not just press the thing, they’re very hard to press I couldn’t use it at all I have no strength in me’*

*(Grace, 78, Telecare standalone, ‘user’)*

A few ‘non-users’ found the equipment inconvenient to use. This was particularly evident across some ‘non-users’ who used the TH standalone equipment. Arthur (92), for example found having to telephone through the readings in the morning inconvenient. Arthur disclosed that given his age he often did not feel good first thing in the morning and so having to provide readings at such an early time was difficult for him. Margaret (75) also found the timings inconvenient. Margaret revealed that the timing of telephoning the readings through did not fit well in her routine. For example, she was up too early to provide the readings but then when she was supposed to do at the allocated time she would often be out.

*‘I don’t agree with the fact that I’ve got to ring up every morning for a few weeks and the chap said “oh no it could be months…I don’t think I would continue to use it because you have to ring up every morning, at a decent time in the morning and I’m not good in the morning you know 8–9 o’clock time in the morning. Damn it I’m 92 years old and you don’t feel good first thing in the morning and you don’t know how long that’s going to last’*

*(Arthur, 92, Telehealth standalone, withdrawn ‘non-user’)*

*‘I thought I would be able to, erm, phone in to check my pulse, erm, I used to read it on the monitor for the pulse as well as the oxygen but, erm, I phoned up every day when I could, but I found it too difficult to phone up every day. I used to end up worrying about it, when I got up it was too early because I was supposed to phone between 9 and 1 and then we went out sometimes and I would forget to phone up’*

*(Margaret, 75, Telehealth standalone, withdrawn ‘non-user’)*

Ken, also found the equipment time consuming and inconvenient. He particularly noted the time-consuming nature of restocking his medication reminder as he had to fill this with his medication four times a day. He also found it inconvenient when he would have to take his medication, for example, if he went out: *‘A little bit of frustration in sorting out so many pill bottles, so many different pills and having to do it three or four times a day. And sometimes if you’re say out in the country having a walk or if you’re in town shopping or whatever, to suddenly think, “Oh, I’ve got to stop and sort out all these pills and get a drink” ‘cause they, it frustratingly inconvenient’* (Ken, 66, Standalone, withdrawn ‘non-user’).

Some of the ‘users’ also shared the frustrations that Ken had around the practicalities of filling up the mediation reminders and taking medication at inconvenient times, which did not necessarily fit around their everyday routine. Many ‘users’ mentioned that it would be really useful if the chemist could restock it for them although this is currently not a service offered by pharmacists:

*‘It’s a bit of a kerfuffle and because I don’t have a regular bedtime and some of those are my bedtime ones you now, not regular down to within the hour. I have to eat before I can take some of these meds and if I don’t get up I don’t want to eat straight away…I hate filling it up because you have everything you take, which Is in that bag’*

*(Sheila, 53, Standalone, ‘user’)*

#### Actual usefulness of equipment

The usefulness of the equipment was viewed as an essential aspect by many of the ‘user/non-user’ participants. In turn, this impacted on the continued engagement with the equipment i.e. if the equipment was viewed useful then patients were more likely to continue to use the service. In particular, if a perceived suitability of the equipment had met a previously unmet need.

The majority of the TC ‘users’ found the equipment useful and felt that the equipment fulfilled a purpose for a number of reasons including; knowing they can get help immediately, reminding them to take their medication and alerting others during emergencies:

*‘Basically its useful to a lot of people, you see people every day that you know you’ll say to them you don’t look well today and I say to them because I’m ill and I know what people look like and I’ll say are you taking your medication? And all of a sudden, they say no I haven’t taken it for two days’*

*(Sue, 46, Telecare standalone, ‘user’)*

*‘The bed sensor is marvelous to me, because I know that I can get in that bed and if I had a seizure, which isn’t very often thank goodness, I know that I am going to be safe, so yeah, it makes me feel safer so it’s very useful, very useful indeed’*

*(Carly, 39, Telecare connected, ‘user’)*

TH ‘users’ found the equipment useful as it enabled them to monitor their health and check that their readings were within the set parameters. This was deemed useful as participants felt reassured and felt they could make a decision to seek health advice. Andrew, a TH ‘user’ reported his heart rate daily to the ATT team who checked his heart rate was within the agreed heart rate parameters. Andrew identified that the usefulness of this equipment was that ATT could have informed him if something is wrong and if he needs to seek medical advice: *‘Well its useful because I know my heart rate, I mean I can see if it is within the set parameters and you know if I need to go to see the doctor they just tell me, before I didn’t know I maybe felt not as good but I guess this provides the reassurance that you are making the right decision’* (Andrew, 66, Telehealth standalone, ‘user’).

However, whilst ‘users’ found the equipment useful there were times where the functionality could be improved. For example, Carole uses a medication reminder but discussed that whilst it was useful when at home when she was out she could not hear it go off: *‘I’ve explained before like if I am in the middle of town when I am out and about and the pill thing is in my bag, sometimes like I say I don’t hear it go off cause of the noise and everything and its only when I get home I think, “Oh god I’ve forgotten”, do you know what I mean, I didn’t hear it’* (Carole, 49, Telecare standalone, ‘user’).

The majority of ‘non-users’ stated that they did not find the equipment useful once they had tried it and in many cases, this was a core reason for non-engagement with the ATT service. Reasons often related to either a lack of need i.e. no perceived need or a change in need:

*‘I didn’t need it anymore, simple as that. The carer went and within two hours of having my plaster cast off I was in hospital so I was looked after there. I only needed it a short time, a week really whilst the carer was here’*

*(Jean. 82. Telecare standalone, withdrawn ‘non-user’)*

*‘I couldn’t see the benefit of it, because I mean I go and have my blood pressure taken once a year at the surgery and you know I have been satisfied with that. I couldn’t see the point, as far as I was concerned, I didn’t see any personal benefit it was wasn’t going to make any difference to my life. I mean what is the benefit of ringing up with my blood pressure. Mines been fairly regular for the past two or three years and I think that’s why I am negative’*

*(Arthur, 92, Telehealth standalone, withdrawn ‘non-user)*

A few ‘non-users’ felt the equipment did not meet their specific requirements and therefore was not useful. Barry, discussed how he wanted to be able to speak to his ‘carer’, which was not an available function on the pager he was using, this ultimately led to his withdrawal of the service: *‘This is a big house and you were a long way from where the carer was sleeping but I couldn’t speak to them on the pager so we ended up texting. It would have been more useful if you could speak to the other person, not useful to us’* (Barry, 59, Telecare standalone, withdrawn ‘non-user’).

Many participants stated that there were ways in which the product design could be improved to make it more useful.

Examples included:

- Having a mechanism on the pressure mat so that it goes off automatically to alert the carer that the patient has got up from bed straight away,
- A GPS watch rather than taking a phone, which the ‘user’ might forget,
- Reduce sensitivity of some of the equipment,
- Improve the medication reminders so that more medication can be stored, making the alarm louder,
- Improving the range of the pendant and pagers,
- Improve usability e.g. larger buttons, removal of difficult catches, making equipment more portable.

#### Functionality of equipment

Functionality related to the operational ability of the equipment, essentially to what extent the ‘user’ could rely and depend on the equipment. Many ‘user/non-user’ participants across the sample felt the equipment was reliable and were confident it would do the job to which it was intended. (Many/some?) TH ‘users’ stated that they knew the readings were accurate and the equipment worked properly:

*‘Yeah its accurate cause I got my own funnily enough, I got my own CPO 2 or whatever it is oxygen thing and I always match cause I calibrate mine with theirs and when I go into hospital I calibrate mine with the machine so I know mines ok, so it must be fairly good equipment’*

*(Steve, 66, Telehealth connected, ‘user’)*

Participants felt happy that it would work during an emergency or when it needed to. This was evident from a wide range of experiences from ‘users’ where they outlined how the equipment worked during situations as expected, was accurate and this reinforced their confidence in the functionality of the equipment:

*‘It’s spot on. Yes, it works very well. Like I’ve got mine set to go off in the evening and often I am driving so it beeps and I have to get off at the next available stop and take my medication but without fail it always goes off’*

*(Roger, 75, Standalone, ‘user’)*

Whilst not all ‘users’ had experienced an emergency, they still felt confident that the equipment would work when it needed to. For example, Mary has Epilepsy and uses both the fall detector and bed sensors. Whilst Mary has not experienced a seizure she still remained confident that if she did have a seizure it would work: *‘I am assuming it would work, I mean I have not had any reason for it to go off yet touch wood but I am confident it will work when it needs to’* (Mary, 85, Telecare connected, ‘user’).

Faulty equipment discussed among the patients centred around medication reminders not releasing medication and coming up with ‘error’, pagers not connecting, TH equipment flickering between readings, loose wire and sensors not working, equipment going off for no reason. However, what appeared to maintain confidence was the excellent support from ATT, who either fixed the faulty equipment and where necessary were given replacements in most cases immediately.

However, whilst ‘non-users’ spoke about having faulty equipment they were not as positive towards the service support. For example, Della discussed how the battery stopped working on her medication reminder after a month of using it. Subsequently, she noticed that a warning sign emerged but was not a battery fault. Della fixed the issue but shortly after, it showed a warning sign again, however, the patient did not know what was wrong with the product and ultimately ceased using the equipment. No contact was made to ATT by Della, as ATT were not aware that they provided the equipment: *‘[T]hen the battery run out and, err, I just left it there I, as going to give it back to the Parkinson’s nurse…to be able to do it seven times, erm, but I’d only it for about a month and all of a sudden it just didn’t work it keep coming up, erm, gold or something. A word that comes up, and I took it to get a battery and they said you don’t need a battery. My son put it right but about 10 min later it just flashed back to this’* (Della, 68, Telecare standalone, withdrawn ‘non-user). Della’s example demonstrates the difficulties of providing a service through the referrer when equipment is not functioning, as it should. The lack of connection patients make between the ATT service equipment and service support is problematic as it may be misunderstood by the patient that the equipment was supplied by their health care professional.

#### Threat to identity and independence

Some ‘user’ and ‘non-user’ patients felt that using the equipment made them feel dependent, having to rely on family and friends as well as a perception of being viewed as helpless. For example, Jim, talks about how he has been fully independent for his whole life, but following the deterioration of his COPD, he was unable to work. He now feels that using assistive technology was just another way of him feeling a ‘burden’ to others: *‘I have worked my whole life but I had a thing about I don’t want to be a burden on society, that’s why for two years I didn’t claim a bloody thing, but it got to a point when savings just dwindle away, using this stuff makes me feel a burden, everyone tells me that I’ve been paying into the system but that doesn’t make me feel better’* (Ian, 69, withdrawn ‘non-user’).

There was agreement across the participants that the equipment created a stigma of ‘being old’ and ‘dependent’. However, as Kelly stated, using the equipment was not just associated with being old but was a sign of a loss of individual independence and an acceptance that life in general would change: *‘It’s not wanting to think that you fall into that elderly category of people. It’s awful. It’s not wanting to admit needing help but it’s not necessary just an age group thing, even elderly don’t like to admit they are getting older and that they need help and things and it’s yeah I think particularly when you have been independent at things, a psychological thing of acceptance’* (Kelly, 66, Telecare connected, ‘user’).

It was also felt by a few ‘users’ that the use of the equipment led to a perceived stigma, whereby, ‘users’ speak of feeling too ‘embarrassed’ to take their equipment out in public in case others see what they are using and will make assumptions of the individual. This was particularly evident across ‘users’ of medication reminders, which were found to be particularly ‘big’ and ‘bulky’:

*‘You get embarrassed because you don’t want to take it out cause people will think “oh you’re taking all those tablets”’*

*(Carole, 49, Standalone, ‘user’)*

*‘I wouldn’t want to take it out with me, if I do go out for the day I just take the tablets I need in my handbag because you don’t want to be in the middle of Marks and Spencer’s and pull this thing out’*

*(Sheila, 53, Standalone, ‘user’)*

## Discussion

This study explored the barriers and facilitators to the initial adoption at the point of referral to the ATT service and the continued engagement of an integrated assistive telehealth and telecare service.

The perceived and actual usability of the equipment impacted on patients’ decisions to adopt and engage with the ATT service. The usability of the equipment is a widely cited reason that has been linked to continued engagement for both TC and TH [32, 57]. This supports the theoretical assumptions of the integrated UTAUT, which posits that effort expectancy directly predicts intention to use technology based devices [36, 37]. In many of the interviews ‘non-users’ stated that they lacked experience and confidence with using the equipment and would have valued more support. Particularly challenging times for patients using equipment, as found in previous research, surrounded the set up and technical problems which included; faulty equipment, inaccurate readings and issues sending the data [25]. The present study supports the wider literature in emphasising the importance of providing support with both prospective and current patients [20]. Effective communication to manage expectations and misconceptions around the difficulty of equipment use reinforces the importance of providing regular individual support to ensure that any technical issues are dealt with in a timely way to enhance continued engagement.

This research also uncovered the influential role of referrers in aiding patients to make decisions about adopting the service. For example, most patients who agreed to use the service made the decision with the referrer who was often a GP or a healthcare specialist. It was found that those who were provided information and made an informed decision about being referred were more likely to agree to use the service. Focus should be placed upon improving the culture for working collaboratively with referrers, with the need to present clear evidence of how such applications will improve health and social care outcomes [58]. More evidence is needed to determine good practice of developing such relationships and will add valuable insights into the development of improving access to integrated services.

There were some ‘non-users’ who withdrew from using TH equipment as they found sending daily readings inconvenient. In contrast, some studies have shown that patients using TH have perceived it to be convenient as it has avoided unnecessary GP and emergency visits [59, 60]. TH patients (65 years and younger) who were able to provide readings online found the device more convenient. Further, in particular for older patients who were able to incorporate time as part of their daily routine to provide their readings over the telephone found it convenient and often stated that they enjoyed the interaction with the staff. Discussions between the patient and provider should therefore consider the patient’s routine and preferences, and where possible make adjustments e.g. the timings readings have to be sent, the method used to send readings which should subsequently improve engagement and patient experience.

Product development and innovation should remain a core aspect of the industry to ensure that the technological equipment continues to advance and meet the needs of the wider service. For example, younger patients were more critical of the technology used and had higher expectations in terms of product functionality. Therefore, there is an increased need to utilise the most up to date technologies. However, for older patients product development related more to improving accessibility for example reducing design flaws which make the equipment difficult to use e.g. small buttons.

In using TC and TH technology, participants showed that this threatened their independence and felt there was an associated stigma with using some of the equipment. Some standalone devices for example were viewed as bulky, and many patients discussed an element of ‘being embarrassed’ and not wanting to attract attention from others asking what the equipment they had was why they used it. Therefore, product development should ensure that such equipment is not only fit for purpose but is aesthetically pleasing and discrete to ensure patients have more privacy around their condition and use.

### Strengths and limitations

This research provided a wide overview of the facilitators and barriers of both the initial adoption and continued engagement of both TH and TC. There are two main strengths of this research. It is the first study to draw upon the views from not only the perspective of ‘users’ who engaged with the service but includes ‘non-users’ who had declined the service after referral alongside those who initially agreed but subsequently withdrew from the service. By exploring ‘users’ and ‘non-users’ in one study has enabled a richer understanding of the influential factors that impact on decision making at different levels of service engagement. This has also enabled a deeper theoretical understanding of user adoption behaviour in the context of an integrated TH and TC service. A further strength is that as this is an already established service this had meant that participants were recruited purposefully rather than restricted to the confines of a trial, a limitation of previous research [20].

There are however, some limitations that are noteworthy. It is firstly important to note that this research is set within the context of an integrated TH and TC service. Whilst this has provided some important information, which relates to the cultural, contextual and organisational factors that can influence the decisions which surround adoption and engagement, these findings may not be generalizable to different models of TH and TC delivery.

The interviews were not conducted by the research team but instead were completed by a trained research assistant (CH) who had experience in qualitative fieldwork. To overcome this limitation, the research team provided the interviewer (CH) with training which involved refresher interview training, ATT induction of equipment and service delivery, alongside going through the research study in full with special attention paid to the conceptual framework on which the study was based and research tools that had been developed. In addition, the audio recordings of the interviews and the typed transcripts were routinely checked by EC and GR for conceptual and method consistency. Discussions around fieldwork reflections further ensured that in-depth and high quality data were collected.

Given the ages of the participants it would have been useful to assess patient health and computer literacy. Further, this study whilst focused on perspectives of patients, did not explore barriers and facilitators from the perspectives of carers, who often play a key role in the referral and decision making process. This is particularly evident for TC and standalone technologies, which are often used to support the carer rather than the patient. Therefore, future research should consider the influential role they have and uncover the perspectives to examine how such services should tailor their equipment to support not only the patients but their carers also.

## Conclusions

The study has highlighted a number of barriers and facilitators to adoption and engagement of TC and TH, referring to the initial decision to use and continue to use the service. The key barriers found included; lack of information of the service and equipment available; the lack of experience and confidence in using the equipment; stigma to using the equipment and inconvenience of using the equipment. The key facilitators were: positive attitudes toward the equipment; usability and reliability of equipment; making the decision to use the service with the referrer and the equipment met the patient’s needs.

The key barriers to using the service may be overcome by an individually tailored approach with collaboration and dialogue between the patient and referrer, this method has been found to improve patient experience and retention rates. In addition, having a face-to-face discussion with a GP or specialist healthcare provider could improve adoption rates and continued engagement with TC and TH. Focus should be placed upon improving the culture for working collaboratively, with the need to present clear evidence of how TC and TH applications could improve health and social outcomes [55]. In turn, collaborative efforts between referrers and patients could ease the acceptance of equipment, allow patients to discuss their feelings about being referred and provide an opportunity to determine the usefulness of the equipment and how it can meet the needs of the patient.

Better communication between referrers and patients could improve attitudes toward TC and TH equipment. The present study found that a positive attitude toward equipment were more likely to adopt and engage with the service. It was clear that reassurance from the onset was paramount, given the potential for negative feelings toward the technology. The need for services to provide detailed information about the equipment available with ‘hands-on’ demonstrations with a discussion of patient expectations on the support they will need through using the service.

There is a dearth of literature in the area of adoption and continued engagement with TC and TH from the perspectives of ‘users’ and ‘non-users’. Disengagement from TH and TC can present a challenge to mainstreamed services [61]. It is important to understand factors that influence decisions and rights to disengage so that services can improve access and support for ‘users’.

### Consent to publish

Consent has been provided from all participants to publish individual patient data.

### Availability of data and materials

The data from this study will not be made publically available as it contains personal identifiable data. The data from this study is part of a larger dataset that was commissioned by CCS and will be made available via a series of published articles.

## Abbreviations

ATT: Assistive Telehealth and Telecare
CCS: Cambridgeshire Community Services
PIS: participant information sheet
TH: Telehealth
TC: Telecare

## References

[1] Age UK (2015). Later life in the United Kingdom.

[2] UK life expectancy still rising [http://www.nhs.uk/news/2011/03March/Pages/uk-life-expectancy-still-rising.aspx]. Accessed 16th Nov 2015.

[3] Clark M, Goodwin N (2010). Sustaining innovation in telehealth and telecare: WSDAN briefing paper.

[4] UK population projected to hit 70 m by 2027 [http://www.ons.gov.uk/ons/dcp29904_240697.pdf]. Accessed 16th Nov 2015.

[5] ESHCRU (2011). Projections of demand for social care and disability benefits for younger adults in England.

[6] Wittenberg R, Hu B, Comas-Herrera A, Fernandez J-L (2012). Care for older people: projected expenditure to 2022 on social care and continuign health care for England’s older population.

[7] Crawford R, Emmerson C (2012). NHS and social care funding: the outlook to 2021/22.

[8] Department of Health. An overview of Telecare and Telehealth. In*.* London: HMSO; 2009.

[9] Department of Health. Whole system demonstrator programme, headline findings. In*.* London: HMSO; 2011.

[10] Department of Health. A concordat between the Department of Health and the telehealth and telecare industry. In*.* London: HMSO; 2012.

[11] Azari R, Pick JB. Understanding global digital inequality: The impact of government, investment in business and technology, and socioeconomic factors on technology utilisation. In: Proceedings of the 42nd Hawaii International Conference on Systems Sciences. Hawaii; 2009.

[12] Barrett D, Thorpe J, Goodwin N, Barrett D, Thorpe J, Goodwin N (2015). Examining perspectives on telecare: factors influencing adoption, implementation, and usage. Med Device Evid Res.

[13] Medical Subject Headings (MeSH) [http://www.nlm.nih.gov/mesh/]. Accessed 16th Nov 2015.

[14] Botsis T, Demiris G, Pedersen S, Hartvigsen G (2008). Home telecare technologies for the elderly. J Telemed Telecare.

[15] Turner KJ, McGee-Lennon MR (2013). Advances in telecare over the past 10 years. Smart Homecare Technol Telehealth.

[16] Korupp SE, Szdlik M (2005). Causes and trends of the digital divide. Eur Sociol Rev.

[17] Millward, P. The ‘grey digital divide’: Perception, exclusion and barriers of access to the Internet for older people. First Monday. 2003;8(7).

[18] Sugarhood P, Wherton J, Procter R, Hinder S, Greenhalgh T (2014). Technology as system innovation: a key informant interview study of the application of the diffusion of innovation model to telecare. Disabil Rehabil Assist Technol.

[19] Clark J, McGee-Lennon M (2011). A stakeholder-centred exploration of the current barriers to the uptake of home care technology in the UK. J Assist Technol.

[20] Sanders C, Rogers A, Bowen R, Bower P, Hirani S, Cartwright M, Fitzpatrick R, Knapp M, Barlow J, Hendy J (2012). Exploring barriers to participation and adoption of telehealth and telecare within the whole system demonstrator trial: a qualitative study. BMC Health Serv Res.

[21] Telemedicine Defined [http://www.amdtelemedicine.com/telemedicine-resources/telemedicine-defined.html]. Accessed 16th Nov 2015.

[22] Carretero S, Centeno C, Stewart J (2013). Telecare and telehealth for informal carers: a research in 12 member states on their benefits and policy role for the success. International congress on telehealth and telecare: 2013.

[23] Zabada C, Singh S, Munchus G (2001). The role of information technology in enhancing patient satisfaction. Br J Clin Govern.

[24] Taylor J, Oguntuase O, Gorst S, Coates E, Armitage CJ. Does telehealth promote self-care? A qualitative study examining the experience of older people using telehealth for long-term condition management. Int J Integr Care. 2014; 14(8).

[25] Gorst SL, Armitage C, Hawley M, Coates E (2013). Explpring patients beliefs and perceptions about sustained use of telehealth. International Journal of Integrated Care.

[26] Polisena J, Tran K, Cimon K, Hutton B, McGill S, Palmer K, Scott RE (2010). Home telehealth for chronic obstructive pulmonary disease: a systematic review and meta-analysis. J Telemed Telecare.

[27] Davis FD (1989). Perceived usefulness, perceived ease of use, and user acceptance of information technologies. MIS Quart.

[28] Azjen I, Kuhl J, Beckham J (1985). From intentions to action: a theory of planned behaviour. Action control: from cognitions to behaviors.

[29] Mackert M (2006). Expanding the theoretical foundations of telemedicine. J Telemed Telecare.

[30] Werner P, Karnieli E (2003). A model of the willingness to use telemedicine for routine and specialised care. J Telemed Telecare.

[31] Werner P (2004). Willingness to use telemedicine for psychiatric care. Telemed e-Health.

[32] Topacan U, Basoglu N, Daim T (2009). Health information service adoption: case of telemedicine. 42nd Hawaii International Conference on System Sciences: 2009; Hawaii, USA.

[33] Rawstorne P, Jayasuriya R, Caputi P (2000). Issues in predicting and explaining usage behaviors with the technology acceptance model and the theory of planned behavior when usage is mandatory. Proceedings of the Twenty First International Conference on Information Systems.

[34] Taylor S, Todd P (1995). Assessing IT usage: the role of prior experience. MIS Quart.

[35] Venkatesh V, Morris MG, Davis GB, Davis FD (2003). User acceptance of information technology: toward a unified view. MIS Quart.

[36] Kijsanayotin B, Pannarunothai S, Speedie SM (2009). Factors influencing health information technology adoptoin in Thailand’s community health centres: applying the UTAUT model. Int J Med Inform.

[37] Carlsson C, Carlsson J (2006). Adoption of mobile devices/services - searching for the answers with the UTAUT. Proceedings of the 39th Annual Hawaii International Conference: 2006.

[38] Rosenstock IM, Becker MH (1974). Historical origins of the health belief model. The health belief model and personal health behavior.

[39] Kirscht JP, Becker MH (1974). The health belief model and illness behavior. The health belief model and personal health behavior.

[40] Rosenstock IM (2005). Why people use health services. Milbank Q.

[41] Hseih HL, Tsai CH (2013). An empiral study to explore the adotion of telehealth; Helath Belief Model perspective. J Eng Sci Technol Rev.

[42] May C, Finch T, Cornford J, Exley C, Gately C, Kirk S, Jenkings K, Osbourne J, Robinson A, Rogers A. Integrating telecare for chronic disease management in the community: What needs to be done? BMC Heal Serv Res. 2011; 11(131). 10.1186/1472-6963-11-131 PMC3116473 21619596

[43] Goodwin N (2010). The state of telehealth and telecare in the UK: prospects for integrated care. J Integr Care.

[44] Cambridgeshire County Council. Cambridgeshire population estimates: Mid-2013. In*.* Cambridegshire: Cambridegshire County Council; 2014.

[45] 2011 Census, Population Estimates by five-year age bands, and Household Estimates, for Local Authorities in the United Kingdom [http://www.ons.gov.uk/ons/rel/census/2011-census/population-estimates-by-five-year-age-bands--and-household-estimates--for-local-authorities-in-the-united-kingdom/index.html]. Accessed 16th Nov 2015.

[46] National Health Service Act c.49. In*.* London: The Stationery Office; 1977.

[47] National Health Service and Community Care Act c.19. In*.* London: The Stationery Office; 1990.

[48] Mandelstam M (2005). Community care practice and the law.

[49] Bower P, Cartwright M, Hirani S, Barlow J, Hendy J, Knapp M, Henderson C, Rogers A, Sanders C, Bardsley M. A comprehensive evaluation of the impact of telemonitoring in patients with long-term conditions and social care needs: protocol for the Whole Systems Demonstrator cluster randomised trial. BMC Heal Serv Res. 2011; 11(184). 10.1186/1472-6963-11-184 PMC3169462 21819569

[50] Ritchie J, Lewis J (2003). Qualitative research practice: a guide for social science students and researchers.

[51] McCaffery K, Forrest S, Waller J, Desai M, Szarewski A, Wardle J (2003). Attitudes towards HPV testing: a qualitative study of beliefs among Indian, Pakistani, African-Caribbean and White British women in the UK. Br J Cancer.

[52] Rabiee F (2004). Focus-group interview and data analysis. Proc Nutr Soc.

[53] Gale NK, Heath G, Cameron E, Rashid S, Redwood S (2013). Using the framework method for the analysis of qualitative data in multi-disciplinary health research. BMC Med Res Methodol.

[54] Ritchie J, Spencer L, Bryman A, Burgess R (1994). Qualitative data analysis for applied policy research. Analysing qualitative data.

[55] Smith J, Firth J (2011). Qualitative data analysis: the framework approach. Nurse Res.

[56] How does NVivo support the Framework method? [http://www.qsrinternational.com/Support/FAQs/How-does-NVivo-support-the-Framework-method]. Accessed 16th Nov 2015.

[57] McCreadie C, Tinker A (2005). The acceptability of assistive technology to older people. Ageing Soc.

[58] Giordano R, Clark M, Goodwin N (2011). Perspectives on telehealth and telecare learning from the 12 Whole System Demonstrator Action Network (WSDAN) sites.

[59] Cottrell E, McMillan K, Chambers R (2012). A cross-sectional survey and service evaluation of simple telehealth in primary care: what do patients think?. BMJ Open.

[60] Gorst SL, Armitage CJ, Brownsell S, Hawley MS (2014). Home telehealth uptake and continued use among heart failure and chronic obstructive pulmonary disease patients: a systematic review. Ann Behav Med.

[61] Rixon L, Hirani SP, Cartwright M, Beynon M, Selva A, Sanders C, Newman S (2013). What influences withdrawal because of rejection of telehealth - the whole systems demonstrator evaluation. J Assist Technol.

